# Dissolution kinetics of silver nanoparticles: Behaviour in simulated biological fluids and synthetic environmental media

**DOI:** 10.1016/j.toxrep.2022.03.044

**Published:** 2022-04-01

**Authors:** Odwa Mbanga, Ewa Cukrowska, Mary Gulumian

**Affiliations:** a Molecular Sciences Institute, School of Chemistry, University of Witwatersrand, Private Bag X3, Johannesburg, 2050, South Africa; b Toxicology and Biochemistry Department, National Institute for Occupational Health, A divisionof National Health Laboratories, Johannesburg 2000, Gauteng, South Africa; c Water research group, Unit for Environmental Sciences and Management, Northwest University, Private Bag X6001, Potchefstroom, 2520, South Africa

**Keywords:** Silver nanoparticles, Agglomeration, Dissolution kinetics, PH, Synthetic, Biological & Environmental media

## Abstract

Silver nanoparticles offer a wide range of benefits including their application in several fields such as medical, food, health care, consumer, and industrial purposes. However, unlocking this potential requires a responsible and co-ordinated approach to ensure that potential challenges emanating from the use of silver nanoparticles are being addressed. In this study body fluids and environmental media were used to investigate the effects of citrate coated silver nanoparticles (cit-coated AgNPs) to mimic their behaviour in real life situations. Understanding the dissolution kinetics and behaviour of cit-coated AgNPs in simulated biological fluids and synthetic environmental media helps us predict their fate and effects on human health and the environment. The cit-coated AgNPs behaviour significantly varied in acidic and alkaline simulated fluids. Low pH and high ionic strength accelerated the rate and degree of dissolution of AgNPs in simulated fluids. Following exposure to simulated fluids cit-coated AgNPs demonstrated significant changes in agglomeration state and particle reactivity however, the morphology remained unaltered. The slow dissolution rates observed for highly agglomerated cit-coated AgNPs in simulated blood plasma, Gamble’s and intestinal fluids, and freshwater indicate that there is a greater likelihood that the particles will be the cause of the observed adverse effects. In contrast, the fast dissolution rates observed for cit-coated AgNPs in simulated gastric and phagolysosomal fluid and synthetic seawater, the release of the silver ions at a fast rate, will be the cause of their short-term effects.

## Highlights

• Understanding the dissolution kinetics of AgNPs to predict their effects on human health and the environment.
• Low pH and high ionic strength accelerated the rate and degree of dissolution of AgNPs in simulated fluids.
• AgNPs demonstrated significant changes in agglomeration state upon exposure to simulated fluids.
• Fast dissolution rates observed for AgNPs in simulated acidic fluids & will be the cause of their short-term effects.

## Graphical Abstract

**Figure:**
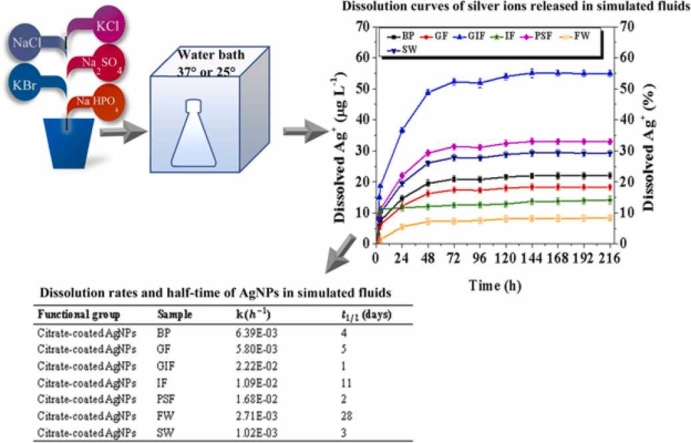

## 1 Introduction

Silver nanoparticles (AgNPs) are extensively used in the nanotechnology industry due to their effective antibacterial activity, high electrical conductivity, and optical properties. They are incorporated in numerous consumer products such as dietary supplements, materials for food packaging, coatings on medical devices, water disinfectants, air filters, electronic appliances, odour-resistant textile fabrics and cosmetic products such as deodorants [3], [4], [5]. Application of AgNPs also includes areas such as biosensing, electronics and surface enhanced Raman spectroscopy [4].

There should be a responsible and co-ordinated approach to ensure that the potential occupational, consumer and environmental safety issues regarding the use of AgNPs are being addressed at the same time as the technology is developing. It is these concerns that have led to a need to elucidate the potential harmful effects of AgNPs to human health and the environment. The assessment of the effects of nanoparticles can be investigated through various properties but in this research study dissolution was employed to determine the effects of nanoparticles on human health and the environment. Dissolution can be explained as the release of species from the nanoparticle surface which leads to the subsequent distribution of those released species throughout the bulk fluid surrounding the nanoparticles. Dissolution heavily influences the biodurability and biopersistence of nanoparticles [41], [9]. The tendency of a given nanoparticle to cause negative effects on human health and the environment is strongly related to the duration of residence time in these environments. Biodurability and persistence are parameters that influence the behaviour of nanoparticles in the body and environment and are used to predict the particles’ duration of residence time under physiological and environmental conditions. For this reason, the dissolution rate is an important property for the assessment of the effects of NPs [8]. Biopersistent nanoparticles are defined as those with the ability to persist in the body due to their biodurability and despite physiological clearance mechanisms [23], [41]. Resistance to physical clearance may result through their resistance to chemical dissolution. In human particle toxicology, the propensity of a nanoparticle to resist alterations through dissolution or enzymatic degradation in biological media is defined as biodurability and could consequently lead to bioaccumulation of nanoparticles in the body following their translocation and distribution [23], [41]. Dissolution also influences the persistence of NPs in the environment. Persistence is defined as the potential of nanoparticles to degrade in the environment. These properties allow for the categorization of nanoparticles between those that are amenable and those resistant to dissolution. For example, particles with slow dissolution rates are expected to elicit long-term effects in human health and the environment. [34], [41].

Numerous dissolution studies of AgNPs reported in the literature have investigated the factors affecting the release of ionic silver under biological and environmental conditions [6,[20], [21], [29], [33], [37], [39]. For example, factors such as pH, ionic strength and surface functionalization have been observed to influence the dissolution of AgNPs where a positive correlation could be observed between low pH conditions and higher dissolution [13], [3], [35], [36]. A positive correlation could also be observed between the salinity of the natural estuarine systems and the higher dissolution of AgNPs [25]. There are other studies which highlight the crucial factor that size and shape have on dissolution of nanoparticles [32]. Despite these numerous studies, there is still a need to investigate the dissolution behaviour of AgNPs in a wide range of simulated biological fluids and environmental media. In addition, many researchers have used a time point greater than 10 min to examine the dissolution behaviour of AgNPs thereby missing crucial information within a key window period during which rapid and major modifications occur to the particles. Furthermore, major focus has been on the highly acidic conditions [36] rather than a wide range of pH conditions to elucidate the dissolution behaviour of AgNPs. Different nanomaterials are reported to be biodurable and persistent as they resist dissolution which in turn can lead to bioaccumulation. It is therefore of crucial importance to investigate the dissolution kinetics of AgNPs in a wide range of simulated biological fluids and environmental media in order to predict their behaviour in real life situations.

Therefore, the present study has investigated the dissolution behaviour and dissolution kinetics (dissolution rates, rate constants and half-times) of citrate coated silver nanoparticles (cit-coated AgNPs) in simulated biological fluids and synthetic environmental media with different chemical composition under a wide variety of pH conditions. Body fluids are very rich in essential elements and may play a very significant role in the intake of NPs and interactions with cellular activities while some are destructive. The body fluids included the Gamble’s fluid (GF) & phagolysosomal fluid (PSF) representing the lung exposure pathway, oral exposure pathway represented by gastric fluid (GIF) and intestinal fluid (IF) and lastly blood plasma (BP). The synthetic environmental media of interest have included Freshwater (FW) and Seawater (SW).

## 2 Materials and methods

### 2.1 Synthesis and characterization of silver nanoparticles

The silver nanoparticles used in this study were purchased from (Sigma Aldrich Johannesburg, South Africa) in the size of 10 nm in diameter suspended in a 1% sodium citrate solution as a stabilizer. Citrate stabilized AgNPs were chosen because citrate is used as a capping agent to maintain the size, monodispersity and stability without irreversible aggregation or decomposition of the silver nanoparticles. Furthermore, citrate stabilized AgNPs are known to be sensitive to changes in pH and the ionic strength of the medium. Therefore, studying their behaviour in vitro would provide an indication of how they would behave in real-life situations. The nanoparticle suspensions were prepared from the stock solution (0.02 mg mL^−1^) under sterile conditions. Transmission Electron microscope (TEM) (JOEL Ltd. JEM-2100) (Lireweg, The Netherlands) was used to obtain information on the structure, size, agglomeration, and level of dispersion of the AgNPs before and after exposure to different simulated biological and environmental fluids. A Bruker Tensor 27 Fourier transform infrared spectroscopy (FTIR) (Billerica, Massachusetts, USA) was used to identify functional groups on molecules attached to NPs and to monitor if they migrated from the nanoparticle surface to the bulk media during the duration of the experiments. The Varian Ultraviolet-Visible 50 Conc. spectrophotometer (UV-is) was used to determine the agglomeration state of AgNPs in simulated media at 400 nm before and after dissolution experiments. The concentration of dissolved silver ions in simulated fluids was determined using inductively coupled mass spectrometer (ICP-MS) (Agilent Technologies, 7700 series ICP-MS, Santa Clara, California, United States).

### 2.2 Procedure

#### 2.2.1 Preparation of fluids

The dissolution experiments were assessed through the use of the static dissolution system whereby the nanoparticle suspension is placed inside a dialysis membrane of a smaller pore size [2,[11], [20], [21], [31], [38]. The sample holder is then submerged in a beaker containing simulated fluids. This methodology employs the fundamental principles of diffusion whereby silver ions will diffuse from a region of high concentration (nanoparticle surface) to that of low concentration (bulk simulated fluid). The rate at which ions are released from the nanoparticle surface is slower than the released ions diffusing out of the dialysis membrane into the bulk media [20], [21]. Therefore, it can be assumed that through measuring the concentration of released ions that passed the dialysis membrane in the bulk fluid by ICP-MS, dissolution kinetics of AgNPs could be predicted.

Simulated biological fluids were synthesized following the procedure obtained from [30] using the anaytical grade reagents described in Table 1. Whereas synthetic environmental media were prepared through the guidelines recommended by the United States (U.S) Environmental Protection Agency (EPA) The focus was on simulated biological fluids intended to mimic the lung, stomach, and intravenous exposure pathways. For the lung, the two main compartments considered were Gamble’s fluid (GF) representing the extracellular airway lining fluid and phagolysosomal fluid (PSF) of macrophage cells at pH 7.4 & pH 4.5, respectively. For the oral route, particles will encounter gastric fluid (GIF)and intestinal fluids (IF) in the stomach at pH 2.0 & pH 7.5 respectively. Lastly, Blood plasma (BP) at pH 7.2 which is a fluid that carries blood components throughout the body. The analytical grade reagents were dissolved in 700 mL of distilled water with a conductivity of 18.2 MΩcm in the order given in Table 1. After addition of all the components the final volume was then adjusted to 1 L. Then the pH was adjusted accordingly using 1 M hydrochloric acid or Sodium hydroxide. For the preservation of simulated fluids the anti-fungal agent alkylbenzyldimethylammonium chloride (ABDC) was added to the fluids at 13.7 mM concentration of (51 µL).

**Table 1:** Composition of simulated fluids [30].

Synthetic fluid	Ionic strength (mol L^−1^)	pH	Components (g L^−1^)
BP^a^	0.15	7.2	NaCl (8.035), NaHCO_3_ (0.355), KCl (0.225), K_2_HPO_4_·3H_2_O (0.231), MgCl_2_· 6H_2_O (0.331), 1 M HCl (39 mL), CaCl_2_ (0.292), Na_2_SO_4_ (0.072), NH_2_C(CH_2_OH)_3_ (6.118)
GF^b^	0.17	7.4	MgCl_2_ (0.203), NaCl (6.019), KCl (0.298), Na_2_HPO_4_ (0.142), Na_2_SO_4_ (0.017), CaCl_2_·2H_2_O (0.368), CH_3_COONa (0.953), NaHCO_3_ (2.604), C_6_H_9_Na_3_O_9_ (0.097)
GIF^c^	0.16	2.0	NaCl (2.922), KCl (7.007), C_8_H_5_O_4_K) (0.243), Pepsin (1 mL mL^−1^), Mucin (3 mg mL^−1^)
IF^d^	0.16	6.8	KCl (0.298), CaCl_2_ (0.499), MgCl_2_ (0.190), Urea (0.300), Bile salts (9 mL mL^−1^), Pancreatin (9 mg mL^−1^)
PSF^e^	0.34	4.5	Na_2_HPO_4_ (0.142), NaCl (6.650), Na_2_SO_4_ (0.072), CaCl_2_·2H_2_O (0.029), Glycine (0.450), C_8_H_5_O_4_K (4.086)
FW^f^	0.05	6.8	NaHCO_3_ (0.012), CaSO_4_ (0.075), MgSO_4_ (0.0075), KCl (0.0005)
SW^g^	3.5	8.0	NaCl (21.03), Na_2_SO_4_ (3.52), KCl (0.61), KBr (0.088), Borax (0.034), MgCl_2_ (9.50), CaCl_2_ (1.320), SrCl_2_ (0.02), NaHCO_3_ (0.17)

Simulated biological fluids and synthetic environmental media are coded as follows:

a BF - Blood plasma,

b GF - Gamble’s fluid,

c GIF - Gastric fluid,

d IF - Intestinal fluid,

e PSF -Phagolysosomal fluid,

f FW - freshwater,

g SW - seawater

### 2.3 Dialysis experiments

Briefly, 5 mL of the cit-coated silver nanoparticle stock suspension (0.02 mg mL^−1^) was pipetted into centrifuge tubes. The particles were collected by ultracentrifugation at 13,000 xg for 30 min. They were then re-dispersed in 5 mL simulated fluids then transferred into dialysis membranes (SnakeSkin Dialysis tubing, 3.5 K MWCO, 22 mm-dry diameter) which were rinsed with d-H_2_O to eliminate any possible contaminants. A smaller pore size membrane was carefully selected to ensure that only released silver ions can migrate from the nanoparticle surface and dialyze out of the tubing. The dialysis experiments were carried out in water baths maintained at 37 °C and 25 °C under slow stirring to mimic physiological conditions and environmental temperature respectively. Furthermore, the experiments were kept under a closed system in an airtight vessel to eliminate background interreferences and from oxidizing species such as oxygen. Therefore, the impact of concentration of dissolved oxygen in the suspensions has not been considered.

The dialysis membranes contained the NP suspensions were submerged in 500 mL beakers containing simulated fluids then placed in a water bath. The dissolution experiments were conducted over a period of 10 days. On the first day samples were collected from the bulk fluid surrounding the dialysis tubing in 30 min intervals for the first four hours, then twice a day for the next 10 days. The pH of the simulated fluids was constantly checked before, during and after each experiment using a Jenway 3510 pH meter. Samples were collected in triplicates and analysed on ICP-MS with a limit of detection of 0.1 ppb. Reported in the results section is an average of the three measurements.

### 2.4 Kinetic model for dissolution process

The determination of the biodurability of nanoparticles using in vitro acellular tests is based on the calculation of their dissolution kinetics. The investigation and determination of the dissolution kinetics and subsequent calculation of dissolution rates and rate constants are in turn, the necessary parameters for the prediction of the nanoparticle biodurability.

Since the dissolution of AgNPs follows a first order reaction kinetics, in current study we modified and used the Noyes-Whitey Eq. (1) to calculate the dissolution kinetics including the dissolution rate and half-time of AgNPs to predict their biodurability in simulated biological and environmental media [20], [21], [42].

(1) $$\frac{d[\mathrm{AgNPs}]}{\mathrm{dt}}=-D\frac{A}{d}({\left[\mathrm{Ag}\right]}_{T}-\;{\left[\mathrm{Ag}\right]}_{\mathrm{dis}})$$

Where $\left[\mathrm{AgNPs}\right]$ denotes the initial concentration of AgNPs stock solution, ${\left[\mathrm{Ag}\right]}_{T}$ represents the concentration of silver before the commencement of dissolution process and ${\left[\mathrm{Ag}\right]}_{\mathrm{dis}}$ represents the concentration of dissolved silver ions. *D* is the diffusion coefficient of silver ions whereas *d* accounts for the diffusion layer thickness during the dissolution process and, *A* represents the surface area of AgNPs.

The total mass of Ag in the system is represented by Eq. 2.

(2) $${\left[\mathrm{Ag}\right]}_{T}=\left[\mathrm{AgNPs}\right]+\;{\left[\mathrm{Ag}\right]}_{\mathrm{dis}}$$

As previously stated, ${\left[\mathrm{Ag}\right]}_{T}$ is the total amount of silver nanoparticles before the dissolution process commences. The $\left[\mathrm{AgNPs}\right]$ then substituted by Eq. 3 in the formula:

(3) $$\left[\mathrm{AgNPs}\right]\;=\;{\left[\mathrm{Ag}\right]}_{T}-\;{\left[\mathrm{Ag}\right]}_{\mathrm{dis}}$$

However, Eq. 3 can also be re-written in the form represented by Eq. 4:

(4) $${\left[\mathrm{Ag}\right]}_{T}-\;{\left[\mathrm{Ag}\right]}_{\mathrm{dis}}=\;{\left[\mathrm{Ag}\right]}_{T}(1-\;\frac{{\left[\mathrm{Ag}\right]}_{\mathrm{dis}}}{{\left[\mathrm{Ag}\right]}_{T}})\;$$

Such that 

(5) $$\left[\mathrm{AgNPs}\right]={\left[\mathrm{Ag}\right]}_{T}(1-\frac{{\left[\mathrm{Ag}\right]}_{\mathrm{dis}}}{{\left[\mathrm{Ag}\right]}_{T}})$$

Eq. 5 is then substituted into Eq. 1 to obtain the following Eq. (6):

(6) $${\left[\mathrm{Ag}\right]}_{T}\frac{d(1-\frac{{\;\left[\mathrm{Ag}\right]}_{\mathrm{dis}}}{{\left[\mathrm{Ag}\right]}_{T}})}{\mathrm{dt}}=\;-D\frac{A}{d}{\left[\mathrm{Ag}\right]}_{T}(1-\;\frac{{\left[\mathrm{Ag}\right]}_{\mathrm{dis}}}{{\left[\mathrm{Ag}\right]}_{T}})$$

Eq. 6 transforms into Eq. 7 if we divide by ${\left[\mathrm{Ag}\right]}_{T}$on both sides of the Eq. 6, yielding the following Eq. 7:

(7) $$\frac{d(1-\frac{{\;\left[\mathrm{Ag}\right]}_{\mathrm{dis}}}{{\left[\mathrm{Ag}\right]}_{T}})}{\mathrm{dt}}=\;-D\frac{A}{d}(1-\;\frac{{\left[\mathrm{Ag}\right]}_{\mathrm{dis}}}{{\left[\mathrm{Ag}\right]}_{T}})$$

For simplicity let $\left(1-\frac{{\left[\mathrm{Ag}\right]}_{\mathrm{dis}}}{{\left[\mathrm{Ag}\right]}_{T}}\right)=x$.

Such that Eq. 7 becomes:

(8) $$\frac{\mathrm{dx}}{\mathrm{dt}}=\;-D\frac{A}{d}x$$

In order to bring x to the left hand-side of the equation and dt to the right hand-side of the equation, Eq. 8 is divided by x and multiplied by dt on both sides of the equation:

(9) $$\;\frac{\mathrm{dx}}{x}=\;-D\frac{A}{d}\mathrm{dt}$$

Upon integration, the above equation becomes:

(10) $$\mathrm{lnx}=\;c-D\frac{A}{d}t$$

Where k = $-D\frac{A}{d}$ and representative of the dissolution rate constant whereas c is a constant.

Eq. 11 is obtained through the integration of Eq. 7.

(11) $$\ln(1-\frac{{\left[\mathrm{Ag}\right]}_{\mathrm{dis}}}{{\left[\mathrm{Ag}\right]}_{T}})\;=\;c-\mathrm{kt}$$

The plot of ln$\left(1-\frac{{\left[\mathrm{Ag}\right]}_{\mathrm{dis}}}{{\left[\mathrm{Ag}\right]}_{T}}\right)$ vs time enables for the determination of the dissolution rate and dissolution rate constant of AgNPs, consequently predicting their biodurability and persistence in biological media and the environment respectively. The half-time for a first order reaction kinetics is calculated using the following formula:

(12) $$t_{1/2}=\frac{\ln2}{k}$$

## 3 Results

### 3.1 Characterization

TEM, UV-is, FTIR were used to assess the morphological changes and agglomeration state of cit-coated AgNPs to draw a link between their dissolution behaviour and physicochemical properties.

#### 3.1.1 TEM

**Fig. 1:**
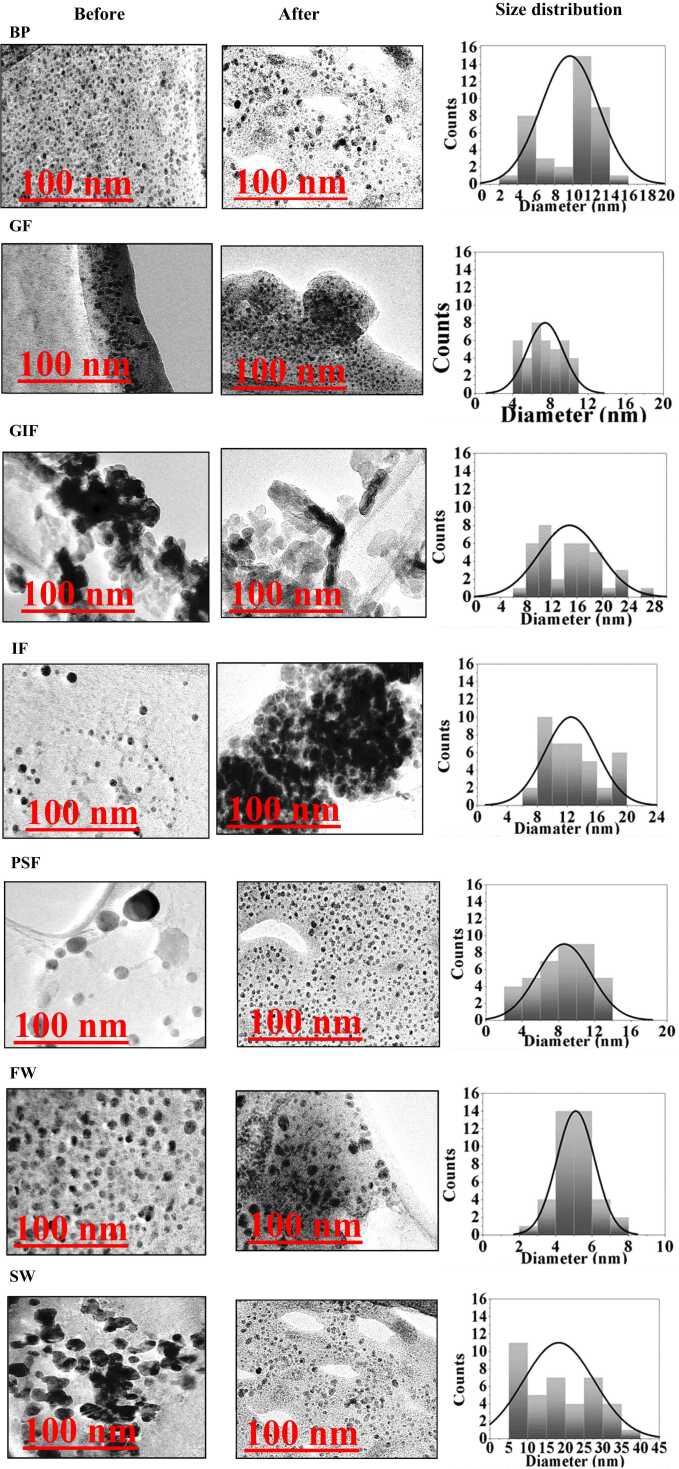
TEM images of cit-coated AgNPs before and after 10 days dissolution experiments in simulated biological and environmental fluids. Simulated biological fluids are coded as BP, GF, GIF, IF and PSF for blood plasma, Gamble’s fluid, gastric fluid, intestinal fluid and phagolysosomal fluid respectively. Whilst synthetic environmental media are denoted as FW and SW are or freshwater and seawater respectively.

TEM images of cit-coated AgNPs before and after 10 days dissolution experiments in simulated biological and environmental fluids. are presented in Fig. 1. The average diameters for cit-coated AgNPs were 10.2 ± 2.1 nm. The size measured from the TEM images were comparable to those reported by the manufacturer to be 10 nm. The nanoparticles were spherical in morphology as seen in the TEM images and there were no appreciable changes in shape after exposure to simulated biological fluids and environmental media (Fig. 1). However, the 10 nm particles showed growth in diameter after dissolution experiments in the simulated intestinal and Gamble’s fluid, blood plasma and freshwater with diameter changing as large as 11.2 ± 3.1 nm compared with original diameters of 10 nm as shown in the TEM images. This might be indicative of formation of nanoparticle agglomerates. In contrast, there was an observable decrease in the size to a diameter as small as 8 ± 2.1 nm of the nanoparticles exposed to acidic media such as simulated gastric and phagolysosomal fluid. This decrease was also observed for the cit-coated AgNPs exposed to seawater which had the highest ionic strength. This decrease likely depicts dissolution in these simulated fluids. These results were corroborated by [29], [3], [36], [40].

#### 3.1.2 UV-vis

**Fig. 2:**
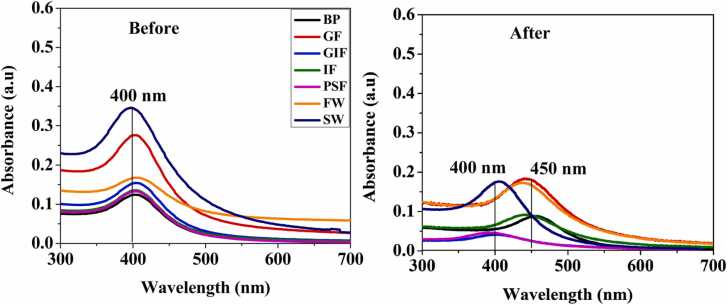
Citrate-coated silver nanoparticle UV–vis absorption spectra before and after the 10 days dissolution experiments in simulated fluids and synthetic environmental media. Simulated biological fluids are coded as BP, GF, GIF, IF and PSF for blood plasma, Gamble’s fluid, gastric fluid, intestinal fluid and phagolysosomal fluid respectively. Whilst synthetic environmental media are denoted as FW and SW are or freshwater and seawater respectively.

Fig. 2 shows the UV–vis spectra of silver nanoparticle dispersion in various simulated fluids and environmental media before and after dissolution experiments to determine the agglomeration state of the particles. For all the simulated fluids before the dissolution experiments, well-defined LSPR peaks can be seen at the wavelength of 400 nm (Fig. 1). This is consistent with the reported LSPR absorption band of silver nanoparticles (Li et al., 2010). However, as the period of exposure to simulated fluids increased there was an observable shift to higher wavelength (450 nm) and a drop in absorption peak intensity for particles in contact with neutral media such as blood plasma, intestinal fluid, Gamble’s fluid and freshwater. For example, a slight shift from 400 nm to 450 nm was observed after dissolution in BP, GF, IF and FW fluids. This indicates that after a prolonged exposure of silver nanoparticles to these simulated fluids the particles physically coalesce to form larger particles. Generally larger particles tend to absorb UV light at higher wavelength than smaller particles hence there was an observable shift to higher wavelengths for the aggregates. This observation is consistent with the TEM images in Fig. 1 that showed formation of larger AgNPs after exposure to these simulated fluids. These results were consistent with those observed by [15]. However, for simulated fluids characterised by low pH (GIF & PSF) and high ionic strength (SW) a decrease in peak intensity and no shift in peak position was observed which could be indicative of particle dissolution. In these fluids, the particles remained dispersed as single NPs hence there was no observable LSPR peak shift to longer wavelengths [45]. These results were consistent with those observed by [1], [13], [16], [17], [27], [44]. There was an observable discolouration of the nanoparticle suspension inside the dialysis membrane from yellow to colourless after exposure to all the simulated fluids.

#### 3.1.3 FTIR

**Fig. 3:**
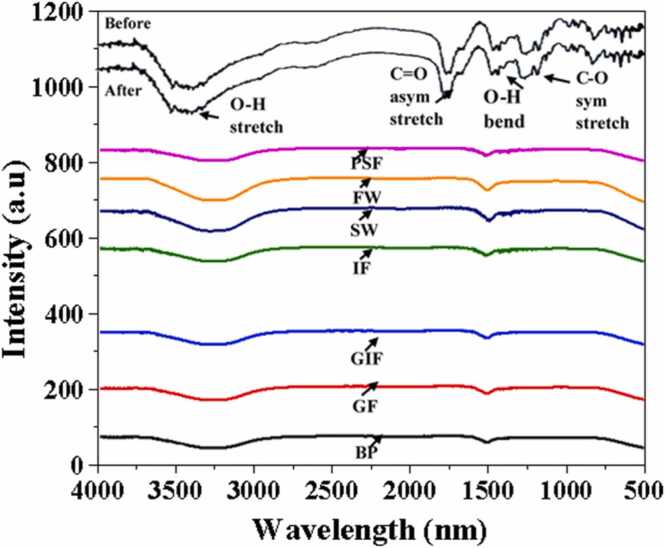
FTIR spectra of cit-coated AgNPs before and after 10 days dissolution and in the bulk fluid after the dissolution process. Simulated biological fluids are coded as BP, GF, GIF, IF and PSF for blood plasma, Gamble’s fluid, gastric fluid, intestinal fluid and phagolysosomal fluid respectively. Whilst synthetic environmental media are denoted as FW and SW are or freshwater and seawater respectively.

FTIR was used to identify functional groups on molecules attached to NPs and to monitor if they migrated from the nanoparticle surface to the bulk media. The absorption wavelength focuses on the range between 500 and 4000 cm^−1^. The cit-coated AgNPs FTIR spectra are shown in Fig. 3. This is the FTIR spectra of silver nanoparticles in the dialysis membrane before exposure to simulated media and after the end of the dissolution experiments, and lastly the bulk fluid outside the dialysis tubing. The absorption peaks bands at 1432 cm^−1^,1619 cm^−1^, 1716 cm^−1^ and 3451 cm^−1^ are assigned to C—O stretch, O—H bend, C=O asymmetric stretch and O-H stretch, respectively.

These intense absorption bands emanate from the symmetric and asymmetric stretching of the carboxylate functional group of the citrate molecules [14], [19], [22], [43]. When comparing the FTIR spectra of the nanoparticle suspension inside the dialysis (black spectra labelled before and after) with those of the bulk fluid outside the dialysis membrane (BP, GF, GIF, IF, PSF, FW and SW graph), there were noticeable differences. The prominent absorption bands which result due to stretching of the citrate functional group were absent. The only observable intense absorption bands were of the O-H stretching of the water molecules used in the synthesis of biological fluids and environmental media. This indicates that no particles migrated from the dialysis membrane into the bulk fluid.

### 3.2 Dissolution in biological fluids and environmental media

**Fig. 4:**
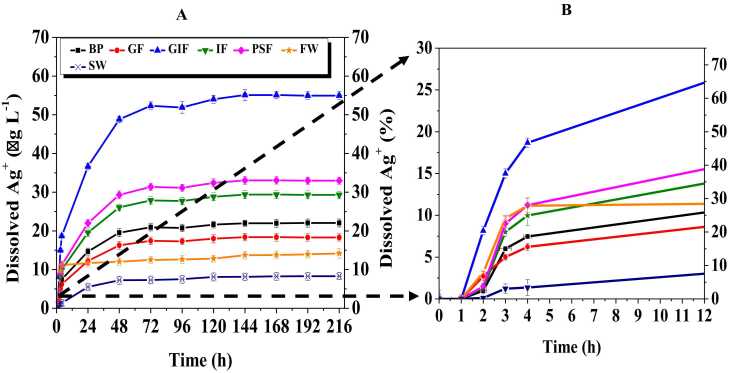
Dissolution curves of silver ions released from the cit-coated AgNPs after exposure to simulated biological and environmental fluids over a period of 216 h (A). Simulated biological fluids are coded as BP, GF, GIF, IF and PSF for blood plasma, Gamble’s fluid, gastric fluid, intestinal fluid and phagolysosomal fluid respectively. Whilst synthetic environmental media are denoted as FW and SW are or freshwater and seawater respectively. Dissolved Ag ions in % was calculated as [Ag(I)]t/[AgNPs]_T_ × 100%. Data only for the initial time point up to 12 h (B).

Fig. 4 shows dissolution curves of cit-coated silver nanoparticles in simulated biological and environmental fluids over a period of 10 days. In all the simulated fluids and synthetic media partial dissolution was observed. This was confirmed by the analysis of the nanoparticle suspension inside the dialysis membrane after the completion of the experiments where nanoparticles were still observed under TEM (Fig. 1). The dissolution of AgNPs increased gradually over time from 4 h then reached a plateau after 120 h. Meaning that it takes less than 24 h for silver ions to interact with the components used in the synthesis of simulated fluids then migrate from the surface of cit-coated AgNPs into the bulk fluid. Significant differences (p < 0.05) were observed in the dissolution trends of cit-coated AgNPs. The concentration of dissolved Ag^+^ ions was smallest for the particles exposed to freshwater. For example, the maximum percentage of dissolved Ag^+^ ion was merely 9.8%. The degree of dissolution depended on the pH and ionic strength of the simulated fluids. Meaning dissolution was higher for simulated fluids characterised by low pH and high ionic strength. For example, the percentage of dissolved Ag^+^ ions were higher for the acidic simulated biological fluids such as gastric and phagolysosomal fluid at 55% and 33%, respectively and high ionic strength seawater at 30% (Fig. 4). This is because under acidic conditions AgNPs are highly unstable therefore likely to dissolve into their ions. Furthermore, the high ionic strength of the sea water significantly favoured the dissolution of AgNPs and shortened their lifetime. Consequently, the percentage of dissolved Ag^+^ ions followed the order GIF > PSF > SW > BP > GF > IF > FW at 55%, 33%, 30%, 22%, 18%, 14% and 9% respectively.

### 3.3 Statistical analysis

The dissolution data shown in Fig. 4 are expressed as mean ± standard deviation of at least three independent measurements. RStudio version 1.2 software was used to process the data and to investigate if there was a statistically significant difference in the dissolution of citrate coated silver nanoparticles in various simulated biological fluids and synthetic environmental media. The p-values are presented in Table 2 and those less than 0.05 were considered statistically significant.

**Table 2:** Multiple variable ANOVA of the dissolution of citrate coated silver nanoparticles in simulated biological fluids and synthetic environmental media.

Nanoparticles	Simulated fluids	*p-value*
AgNPs	BP	0.0021
AgNPs	GF	0.0008
AgNPs	GIF	0.0144
AgNPs	IF	0.0420
AgNPs	PSF	0.0231
AgNPs	FW	0.0320
AgNPs	SW	0.0231

According to multiple variable ANOVA statistical analysis the p values are less than 0.05 in all cases meaning the dissolution of silver nanoparticles differed significantly among the various simulated fluids. Therefore, silver nanoparticles are expected to behave differently in simulated biological fluids and synthetic environmental media.

### 3.4 Dissolution kinetics

**Fig. 5:**
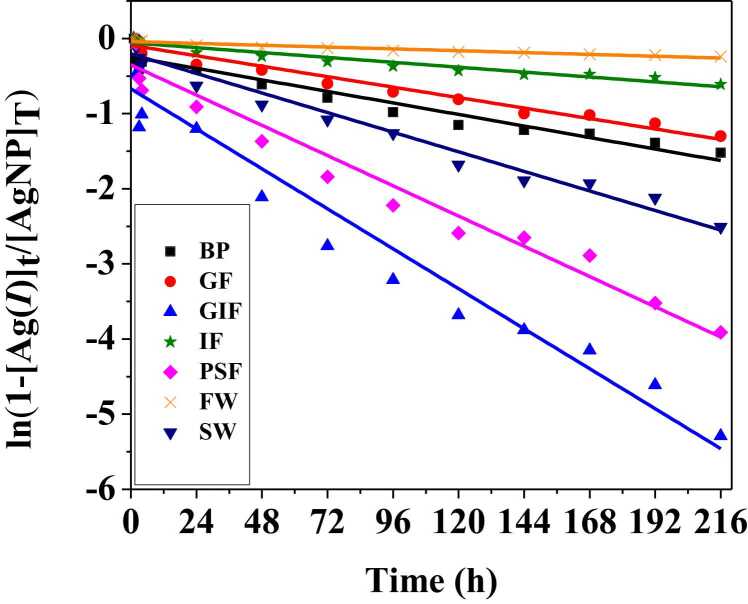
Dissolution rates of cit-coated AgNPs in simulated biological and environmental fluids. Simulated biological fluids are coded as BP, GF, GIF, IF and PSF for blood plasma, Gamble’s fluid, gastric fluid, intestinal fluid and phagolysosomal fluid respectively. Whilst synthetic environmental media are denoted as FW and SW are or freshwater and seawater respectively.

The first order kinetic model shown in the materials and method section was used to predict the dissolution kinetics of silver nanoparticles. This model enables for the calculation of the dissolution rate constant and half-times of silver nanoparticles in simulated fluids in order to predict their residence time in these biological and environmental surroundings. Therefore, Fig. 5 shows the dissolution rates of citrate coated AgNPs in simulated fluids and synthetic environmental media. The results of dissolution rates k (h ^−1^) and the calculated corresponding half-times t_1/2_ (days) are presented in Table 3.

**Table 3:** Dissolution rates and half-time of AgNPs in simulated biological and environmental fluids.

Nanoparticles	Sample	k ($h^{-1}$)	$t_{1/2}$ (days)
AgNPs	BP	6.39E-03	4
AgNPs	GF	5.80E-03	5
AgNPs	GIF	2.22E-02	1
AgNPs	IF	2.71E-03	11
AgNPs	PSF	1.68E-02	2
AgNPs	FW	1.02E-03	28
AgNPs	SW	1.09E-02	3

It was observed that dissolution kinetics depended on the pH and the fastest dissolution was observed for acidic media such as simulated gastric and phagolysosomal fluid whose pH levels are 2.0 and 4.5 respectively. The steeper slope of the linearized model for gastric and phagolysosomal fluid indicate faster dissolution rate (Fig. 5). The dissolution rates were 2.22E-02 h^−1^and 1.68E-02 h^−1^for gastric fluid and phagolysosomal fluid respectively (Table 3). Another factor observed to influence dissolution kinetics was the ionic strength of the media. Comparison of the silver nanoparticle behaviour in environmental media revealed that particles exposed to high ionic strength seawater will dissolve faster than those submerged in freshwater. For example, Table 3 indicates that it will take approximately 3 days for silver nanoparticles to dissolve in sweater whereas the same process would require about 28 days in a freshwater environment. The dissolution half-times followed the order of GIF > PSF > SW > BP > GF > IF > FW at 1, 2, 3, 4, 5, 11 and 28 days, respectively. Lastly, highly agglomerated particles have a reduced surface area thus slow dissolution rates.

Using data obtained from literature the dissolution kinetics of silver nanoparticles were compared with the dissolution rates of various nanoparticles to assess whether silver would elicit short-term or long-term effects on human health and the environment. Table 4.

**Table 4:** Comparison of various nanoparticle dissolution kinetics in simulated fluids.

Nanoparticles	Conditions	k ($h^{-1}$) From literature	$t_{1/2}$ (days) From literature
Synthesised CuO 7 ± 1 nm Commercial CuO 31 ± 4 nm	Simulated body fluid Simulated body fluid pH7.4	0.644 0.469 [9]	
AgNPs 10 nm (Present study)	Blood plasma Gamble’s fluid Gastric fluid Intestinal fluid Phagolysosomal fluid Freshwater Seawater		4 5 1 11 2 28 3
ZnO 63.6 nm	Wastewater effluents pH 7.5		0.12 (h^−1^) [42]
Citrate coated AuNPs 14 nm	Simulated freshwater pH 6.8		853 [31]
BaSO_4_ NM-220 32 nm	Simulated phagolysosomal fluid pH 4.5		6.8 [18]

When comparing the dissolution kinetics of other nanoparticles from literature to that of silver nanoparticles of the present study, it could be observed that zinc oxide nanoparticles have faster dissolution kinetics followed by copper oxide nanoparticles. Barium sulphate nanoparticles also showed faster dissolution because the experiments were carried out in a continuous flow through system which does not reach saturation, and this adds to faster dissolution rates of nanoparticles. Gold nanoparticles were observed to have very low dissolution rates.

## 4 Discussion

Evident from the TEM images and UV–vis spectra of simulated blood plasma, Gamble’s fluid, intestinal fluid and synthetic freshwater was the agglomeration of cit-coated AgNPs after dissolution experiments. Particle agglomeration reduces the surface are to volume ratio of the nanoparticle surface thereby attenuating chances of dissolution. This would explain slow dissolution rates of AgNPs in these simulated fluids. On the other hand, a decrease in the initial particle size of cit-coated AgNPs from 10.2 ± 2.1 nm to 8.1 ± 2.1 submerged in gastric and phagolysosomal fluid and synthetic seawater might have resulted from the dissolution of AgNPs as a result of low pH and high concentration of chloride ions in seawater. According to the Noyes-Whitney equation dissolution of nanoparticles is directly proportional to the particle surface area, as a result monodispersed nanoparticle should dissolved faster than those characterized by agglomeration. Smaller particles have a larger surface area that could release ions faster hence, they provide greater dissolution than larger particles. This might explain the higher dissolution of cit-coated AgNPs immersed in low acidic pH gastric and phagolysosomal fluid and high ionic strength synthetic seawater. These results were consistent with those observed by [7]. [26] investigated dissolution and aggregation of silver nanoparticles in environmental media and observed a positive correlation between high dissolution and low acidic pH conditions. Peretyazhko et al. [36] stated that when AgNPs are exposed to media, the formation of silver oxide on the nanoparticle surface initiates dissolution. This in turn releases silver Ag^+^ ions into the solution until the silver oxide complex is completely dissolved. Furthermore, low acidic conditions such as gastric and phagolysosomal fluid have a higher abundance of protons which weaken the Ag-O bond resulting in the release of more Ag^+^ ions. This might explain the higher dissolution of cit-coated AgNPs immersed in low acidic pH gastric and phagolysosomal fluid.

Furthermore, the high dissolution of AgNPs in seawater could be due to the high concentration of chloride ions which characterise this synthetic environmental media. Chloride is a hard base ligand with a strong affinity for oxidized silver [24]. Therefore, Li et al. [25] and Li et al. [28] have indicated that the dissolution of AgNPs is highly influenced by the ratio between Ag/Cl in a solution. Meaning if a solution has a high concentration of free Cl^-^ ions as is the case with seawater, then chances are that the chloride ions will interact with Ag^+^ ions forming more soluble complexes (AgCl) thereby encouraging dissolution. Owing to the high concentration of chloride ions in synthetic seawater, the formation of AgCl complexes is more pronounced. Therefore, the AgCl bridging between AgNPs might reduce AgNPs-AgNPs agglomeration leaving the particles monodispersed with a more exposed surface area thus increasing the chances of dissolution [10] corroborates these results. Furthermore, these results are consistent with previous research observation that have suggested that chloride ions might act as a catalyst in the dissolution of AgNPs [10].

It therefore seems that both low acidic solutions and higher ionic strength could result in lower particle agglomeration leading to higher dissolution of cit-coated AgNPs.

## 5 Conclusion

In this study the potential impact of AgNPs on human health and the environment were investigated through studying their dissolution. It could be shown that Cit-coated AgNPs form agglomerates when introduced to alkaline simulated fluids and this could reduce the surface area thereby lowering chances of dissolution. The rate and degree of the dissolution of cit-coated AgNPs is influenced by pH and ionic strength of simulated fluids. The dissolution rates of particles immersed in simulated gastric fluid, phagolysosomal fluid and seawater were faster compared to the rates of particles exposed to more alkaline biological and environmental media. Meaning that silver nanoparticles in acidic media and fluids characterized by high ionic strength would exhibit short-term effects which could be identical to those of the dissolved ions. However, the potential health and environmental impact of AgNPs in blood plasma, Gamble’s & intestinal fluids and freshwater would be expected to be much worse.

## CRediT authorship contribution statement

**Odwa Mbanga**: Conceptualization, Methodology, Validation, Formal analysis, Investigation, Writing – original draft. **Ewa Cukrowska**: Resources, Writing – review & editing, Visualization. **Mary Gulumian**: Resources, Writing – review & editing, Visualization, Supervision, Funding acquisition.

## Declaration of Competing Interest

The authors declare that they have no known competing financial interests or personal relationships that could have appeared to influence the work reported in this paper.
